# Resistance Training in Face of the Coronavirus Outbreak: Time to Think Outside the Box

**DOI:** 10.3389/fphys.2020.00859

**Published:** 2020-07-07

**Authors:** Paulo Gentil, Rodrigo Ramirez-Campillo, Daniel Souza

**Affiliations:** ^1^College of Physical Education and Dance, Federal University of Goiás, Goiânia, Brazil; ^2^Hypertension League, Federal University of Goias, Goiânia, Brazil; ^3^Laboratory of Human Performance, Quality of Life and Wellness Research Group, Department of Physical Activity Sciences, Universidad de Los Lagos, Osorno, Chile

**Keywords:** COVID19, human physical conditioning, virus infection, exercise is medicine, barbell, dumbbell, muscle strength, weightlifting

## The Problem

Resistance training (RT) is an exercise type commonly associated with the performance of muscle contractions against external resistance. This training model is very popular and has been recommended as an essential part of an exercise program by several important associations (ACSM, 2009; Garber et al., 2011). Its benefits are commonly associated with muscle strength and mass gains and expand to several areas such as blood pressure control (MacDonald et al., 2016), improved bone mineral density (Zhao et al., 2015), depression management (Gordon et al., 2018), cancer treatment (Fuller et al., 2018), controlling blood glucose (Codella et al., 2018), weight management (Paoli et al., 2014), among others. Such benefits, largely mediated by strength gains, culminate in reductions in mortality rates in different populations (Ruiz et al., 2008; Artero et al., 2011; Ortega et al., 2012; Hardee et al., 2014; Dankel et al., 2016). Many of the problems that RT has been shown to counteract are related to increased mortality and morbidity associated with COVID, like hypertension, diabetes, coronary diseases, overweight (Muniyappa and Gubbi, 2020; Salerno et al., 2020; Shahid et al., 2020; Singh et al., 2020; Zhou et al., 2020). Although it is not possible to attribute a direct cause-effect relationship between RT and mortality risk during COVID, it might be important to perform RT to improve general health and help in a better prognostic in case of contamination.

Notwithstanding, in the face of the coronavirus or COVID-19 outbreak, the practice of RT has been strongly threatened. Considering that this virus is highly contagious and may be transmitted by close contact among people and by sharing subjects, public health policies generally recommend distance from other people, avoidance of tight spaces and agglomeration as preventive measures (Adhikari et al., 2020). Therefore, RT practice faces new challenges and these should be addressed in the near future, for practical and logistical reasons. However, this might not be an inherent problem to RT, but rather in the way that it is commonly understood and applied. Although it originally involved exercises with body weight or objects obtained from the nature itself, RT began to be increasingly associated with sophisticated equipment and facilities, and with time-consuming, and complicated routines. As a result, RT is being mainly performed in facilities such as gyms, health clubs, fitness centers, and with high-cost specialized machines. Such conceptions regarding RT imposes a barrier for its implementation in the current scenario, since its performance would involve agglomeration, climatized environments, sharing of materials, and other characteristics that may favor an increased risk of infection. However, is it important to note that RT does not necessarily involve the need of conventional equipment and facilities which allows it to be performed in a range of alternative situations.

When analyzing current scientific evidence, it seems that RT can safely, time-efficient and easily be implemented, in almost anywhere and with minimal resources, which makes it fully feasible within measures adopted to control coronavirus dissemination. Therefore, this opinion aimed to discuss practical and uncomplicated evidence-based RT alternatives to overcome the restrictions measures adopted during COVID-19 outbreak.

## Loads, Equipment and Implements

Most conventional approaches suggest that RT should be performed with moderate to high loads, with specific number of repetitions and using specific equipment, like machines and free weights (Kraemer et al., 2002; ACSM, 2009). However, according to the muscle effort principle (i.e., effort-based paradigm; Steele et al., 2017a), when effort is high, RT performed in different ways, such as using different loads, different types of equipment and in different environments, can bring gains in muscle size and fitness similar to the most conventional approaches (Fisher et al., 2017b; Steele et al., 2017b, 2019).

With regard to load, it is commonly recommended that, for optimal gains in strength and muscle mass, it is necessary to use moderate- to high-loads [≥60% of one repetition maximum (1RM)] (McDonagh and Davies, 1984; ACSM, 2009), which would make it difficult to perform RT without specialized equipment. However, studies have shown that, regardless of training level, gains in muscle strength, and mass may be related to muscle effort (i.e., physiological stimulus), and not necessarily to the load being used (Fisher et al., 2017b).

Studies in untrained (Mitchell et al., 2012; Assunção et al., 2016) and trained people (Morton et al., 2016) reveal that training with low-external loads (LEL) promote similar gains in muscle mass and strength compared to high-external loads, especially when the strength tests are not specific (Fisher et al., 2017b). In fact, relevant gains in strength and muscle mass are feasible with LEL (40% of 1RM) and a high number (>100) of repetitions (Farup et al., 2015), something that was previously unimaginable for RT. Although the studies with LEL involved the precise quantification of the load, which might not be feasible during the social distance measures, the results suggested that RT benefits might be associated with effort and not with the number of repetitions or loads used. Therefore, it brought the insight that muscles do not “see” the load being lifted or count repetitions performed, but it seems to interpret physiological signaling associated with effort (Steele et al., 2017a, 2019). With this in mind, it might be expected that training with affordable implements (elastic bands, light dumbbells, and barbells) without the need of specialized resources or facilities could be a feasible option for RT.

Indeed, similar physiological stimulus can be induced with elastic bands when compared to traditional methods, including muscle activation and micro-structural damage (Aboodarda et al., 2011, 2016), strength gains (Martins et al., 2013), and functional improvements (Colado et al., 2010; Souza et al., 2019). Moreover, some training models traditionally associated with aerobic activities such as stationary cycling may promote muscle hypertrophy and strength gains (Ozaki et al., 2015, 2016; Steele et al., 2019). Such a proposal has already been presented and tested, with promising results (Steele et al., 2019). Therefore, effective RT programs can be performed using implements easily obtained in standard commercial facilities at low cost, and that might be stored at home.

Moreover, significant peripheral physiological stimulus can be induced even without external-load (NEL), involving maximal or near-maximal voluntary muscle contraction. For example, acute studies verified high levels of motor units recruitment when performing NEL muscle contractions with the intention to maximally contract the muscles (Gentil et al., 2017a). Some studies showed muscle strength and mass gains after NEL-RT programs (Counts et al., 2016; Barbalho et al., 2019). In young men and women, after a contralateral training design, equivalent gains in the arm muscle size was observed after traditional RT and NEL-RT (Counts et al., 2016). This hypothesis was also confirmed in rehabilitation setting, with positive outcomes in terms of hypertrophy and functionality (Barbalho et al., 2019).

Therefore, evidence points toward the need of inducing significant muscle physiological stimulus and this can be achieved with LEL, or even with NEL. Therefore, the need for equipment and implements should not be viewed as a barrier to implement RT programs during current pandemic times. On the other hand, due the high levels of perceived effort and discomfort during NEL and LEL (Fisher and Steele, 2017; Gentil et al., 2017a), as well as the high cardiovascular stress induced by LEL (Vale et al., 2018), is reasonable to suggest that these training methods might not be suitable for special population, such as sedentary aging people and heart disease patients.

Finally, RT has been usually monitored by external load parameters, which might not be feasible in absence of specialized equipment. However, it has been suggested that that monitoring internal load might be also important to understand exercise response (Impellizzeri et al., 2019). In this regard, session rate of perceived effort and training impulse (repetitions x session rate of perceived exertion) might be useful tools for this purpose (Martorelli et al., 2020). According to (Martorelli et al., 2020), session rating of perceived exertion might be more indicated when training to or close to muscle failure, while training impulse might be indicated when avoiding muscle failure.

## Time-Efficiency and Uncomplicated Training Strategies

A practical issue that raised with social isolation was the reduction of free time. Although social isolation may presume more time available it is also possible that greater involvement in household activities, family care and children's education, for example, might make time more scarce. Thus, the need for time-efficient RT approaches has become promising and attractive (Fisher et al., 2017a). If we consider a minimum dose approach, workouts that last a few minutes can be efficient in promoting muscle strength and size gains (Souza et al., 2020).

Another important aspect is about exercise selection. It is commonly believed that a complete routine would require a wide variety of exercises, including many isolated exercises for specific muscles, like gluteus, biceps, and triceps brachii. However, current evidence shows that the use of basic and multi-joint exercises is sufficient to promote gains in muscle strength and size in most muscles involved in movement (Gentil et al., 2015, 2017b; Paoli et al., 2017; Barbalho et al., 2020a,b) and the addition of isolated exercises, in general, does not seem to bring additional benefits (Gentil et al., 2013; de França et al., 2015; Barbalho et al., 2020b). Furthermore, RT using body weight promotes similar gains in muscle strength and thickness in comparison with traditional training, even in young trained practitioners (Calatayud et al., 2015; Kikuchi and Nakazato, 2017), as: provides benefits to middle-aged people with non-alcoholic fat liver disease (Takahashi et al., 2015, 2017) and improvements on muscle strength and body composition in elderly people (Tsuzuku et al., 2017). Although the study of Kikuchi and Nakazato (2017) had a limited sample size and a high variability in the results, it is possible that body-weight exercises (such as jumps, dips, push-ups, chin-ups, squats, and lunges) performed at home, requiring no extra equipment, in a relatively reduced space, with a low-volume time-efficient approach can be capable of inducing significant health benefits and increases in muscle strength, power, and hypertrophy.

A common belief related to RT is the need of constant exercise variation to guarantee continued results over medium and long-term. This might lead to the misconception that it would not be possible to obtain positive results unless several different types of equipment and loads are available, as in traditional facilities. However, variation of external load, methods and exercises do not necessarily translates into greater muscle strength and hypertrophy gains (Loturco and Nakamura, 2016; Loturco et al., 2016; De Souza et al., 2018; Baz-Valle et al., 2019; Damas et al., 2019). In this sense, proper adjustment of RT intensity and volume in order to provide an adequate muscle physiological stimulus might be more beneficial to achieve optimum gains in muscle strength and size, than to vary the RT program *per se*.

## Final Considerations

The repercussions of the current pandemic on the health of economy is a major concern for most countries around the globe. With people at home, sedentary behavior, and physical inactivity may increase, posing an even greater burden to the economy (Ding et al., 2016) and increasing the prevalence of problems associated with greater mortality and morbidity associated with COVID-19. Home-based (or public open spaces) RT programs in the context of current pandemic times are highly feasible, with an enormous potential impact in counteracting the detrimental effects of sedentary behavior and physical inactivity on population health and economic burden.

Several effective, safe, low-cost, time-efficient, and practical RT approaches can be considered, such as programs with LEL, NEL, body-weight load, jump-based, elastic bands, among others. Complicated, highly specialized exercises are not an essential part of RT programs for most people. RT programs should be promoted during current pandemic times in order to promote better health and quality of life (Tasiopoulos et al., 2018; Nikolaidis et al., 2019). However, it might be necessary to sacrifice some old-fashioned thoughts rooted in beliefs that have already been overturned by science. Analyzing the new perspectives brought in this opinion article, we see that it is possible to perform RT in a practical and uncomplicated manner, avoiding the exposure to a potentially infectious environment and adapting training programs to the new time and logistical challenges brought by the coronavirus outbreak. These strategies might allow obtaining continued results in terms of mental and physical health. These seems be the returning message of to what Leonardo Da Vinci told us more than 500 years ago: “simplicity is the ultimate sophistication.”

## Author Contributions

PG, RR-C, and DS contributed to the conception, drafting the article, revising it critically, and final approval of the version to be published. All authors contributed to the article and approved the submitted version.

## Conflict of Interest

The authors declare that the research was conducted in the absence of any commercial or financial relationships that could be construed as a potential conflict of interest.

## References

[B1] Aboodarda S. J. George J. Mokhtar A. H. Thompson M. (2011). Muscle strength and damage following two modes of variable resistance training. J. Sport. Sci. Med. 10, 635–642. 24149552 PMC3761515

[B2] Aboodarda S. J. Page P. a. Behm D. G. (2016). Muscle activation comparisons between elastic and isoinertial resistance: a meta-analysis. Clin. Biomech. 39, 52–61. 10.1016/j.clinbiomech.2016.09.008 27681867

[B3] ACSM (2009). American College of Sports Medicine position stand. Progression models in resistance training for healthy adults. Med. Sci. Sport. Exerc. 41, 687–708. 10.1249/MSS.0b013e3181915670 19204579

[B4] Adhikari S. P. Meng S. Wu Y. J. Mao Y. P. Ye R. X. Wang Q. Z.. (2020). Epidemiology, causes, clinical manifestation and diagnosis, prevention and control of coronavirus disease (COVID-19) during the early outbreak period: a scoping review. Infect. Dis. Poverty 9, 1–12. 10.1186/s40249-020-00646-x 32183901 PMC7079521

[B5] Artero E. G. Lee D. C. Ruiz J. R. Sui X. Ortega F. B. Church T. S.. (2011). A prospective study of muscular strength and all-cause mortality in men with hypertension. J. Am. Coll. Cardiol. 57, 1831–1837. 10.1016/j.jacc.2010.12.025 21527158 PMC3098120

[B6] Assunção A. R. Bottaro M. Ferreira-Junior J. B. Izquierdo M. Cadore E. L. Gentil P. (2016). The chronic effects of low- and high-intensity resistance training on muscular fitness in adolescents. PLoS ONE 11:e160650. 10.1371/journal.pone.0160650 27509050 PMC4979886

[B7] Barbalho M. Coswig V. Souza D. Serrão J. C. Campos M. H. Gentil P. (2020a). Back squat vs. hip thrust resistance-training programs in well-trained women. Int. J. Sports Med. 41, 306–310. 10.1055/a-1082-1126 31975359

[B8] Barbalho M. Coswig V. S. Bottaro M. De Lira C. A. B. Campos M. H. Vieira C. A.. (2019). “nO LOAD” resistance training increases functional capacity and muscle size in hospitalized female patients: a pilot study. Eur. J. Transl. Myol. 29, 302–306. 10.4081/ejtm.2019.8492 31908746 PMC6926436

[B9] Barbalho M. Souza D. Coswig V. Steele J. Fisher J. Abrahin O.. (2020b). The effects of resistance exercise selection on muscle size and strength in trained women. Int. J. Sports Med. 10.1055/a-1121-7736. [Epub ahead of print]. 32252103

[B10] Baz-Valle E. Schoenfeld B. J. Torres-Unda J. Santos-Concejero J. Balsalobre-Fernández C. (2019). The effects of exercise variation in muscle thickness, maximal strength and motivation in resistance trained men. PLoS ONE 14, 1–10. 10.1371/journal.pone.0226989 31881066 PMC6934277

[B11] Calatayud J. Borreani S. Colado J. C. Martin F. Tella V. Andersen L. L. (2015). Bench press and push-up at comparable levels of muscle activity results in similar strength gains. J. Strength Cond. Res. 29, 246–253. 10.1519/JSC.0000000000000589 24983847

[B12] Codella R. Ialacqua M. Terruzzi I. Luzi L. (2018). May the force be with you: why resistance training is essential for subjects with type 2 diabetes mellitus without complications. Endocrine 62, 14–25. 10.1007/s12020-018-1603-7 29730785

[B13] Colado J. C. Garcia-Masso X. Pellicer M. Alakhdar Y. Benavent J. Cabeza-Ruiz R. (2010). A comparison of elastic tubing and isotonic resistance exercises. Int. J. Sports Med. 31, 810–817. 10.1055/s-0030-1262808 20703977

[B14] Counts B. R. Buckner S. L. Dankel S. J. Jessee M. B. Mattocks K. T. Mouser J. G.. (2016). The acute and chronic effects of “NO LOAD” resistance training. Physiol. Behav. 164, 345–352. 10.1016/j.physbeh.2016.06.024 27329807

[B15] Damas F. Angleri V. Phillips S. M. Witard O. C. Ugrinowitsch C. Santanielo N.. (2019). Myofibrillar protein synthesis and muscle hypertrophy individualized responses to systematically changing resistance training variables in trained young men. J. Appl. Physiol. 127, 806–815. 10.1152/japplphysiol.00350.2019 31268828

[B16] Dankel S. J. Loenneke J. P. Loprinzi P. D. (2016). Determining the importance of meeting muscle-strengthening activity guidelines: is the behavior or the outcome of the behavior (strength) a more important determinant of all-cause mortality? Mayo Clin. Proc. 91, 166–174. 10.1016/j.mayocp.2015.10.017 26723715

[B17] de França H. S. Branco P. A. N. Guedes Junior D. P. Gentil P. Steele J. Teixeira C. V. L. S. (2015). The effects of adding single-joint exercises to a multi-joint exercise resistance training program on upper body muscle strength and size in trained men. Appl. Physiol. Nutr. Metab. 826:150409143403004 10.1139/apnm-2015-0109 26244600

[B18] De Souza E. O. Tricoli V. Rauch J. Alvarez M. R. Laurentino G. Aihara A. Y.. (2018). Different patterns in muscular strength and hypertrophy adaptations in untrained individuals undergoing nonperiodized and periodized strength regimens. J. Strength Cond. Res. 32, 1238–1244. 10.1519/JSC.0000000000002482 29683914

[B19] Ding D. Lawson K. D. Kolbe-Alexander T. L. Finkelstein E. A. Katzmarzyk P. T. van Mechelen W.. (2016). The economic burden of physical inactivity: a global analysis of major non-communicable diseases. Lancet 388, 1311–1324. 10.1016/S0140-6736(16)30383-X 27475266

[B20] Farup J. de Paoli F. Bjerg K. Riis S. Ringgard S. Vissing K. (2015). Blood flow restricted and traditional resistance training performed to fatigue produce equal muscle hypertrophy. Scand. J. Med. Sci. Sport. 25, 754–763. 10.1111/sms.12396 25603897

[B21] Fisher J. Steele J. Smith D. (2017b). High- and low-load resistance training: interpretation and practical application of current research findings. Sport. Med. 47, 393–400. 10.1007/s40279-016-0602-1 27480764

[B22] Fisher J. P. Steele J. (2017). Heavier and lighter load resistance training to momentary failure produce similar increases in strength with differing degrees of discomfort. Muscle Nerve 56, 797–803. 10.1002/mus.25537 28006852

[B23] Fisher J. P. Steele J. Gentil P. Giessing J. Westcott W. L. (2017a). A minimal dose approach to resistance training for the older adult; the prophylactic for aging. Exp. Gerontol. 99, 80–86. 10.1016/j.exger.2017.09.012 28962853

[B24] Fuller J. T. Hartland M. C. Maloney L. T. Davison K. (2018). Therapeutic effects of aerobic and resistance exercises for cancer survivors: a systematic review of meta-analyses of clinical trials. Br. J. Sports Med. 52:1311. 10.1136/bjsports-2017-098285 29549149

[B25] Garber C. E. Blissmer B. Deschenes M. R. Franklin B. A. Lamonte M. J. Lee I. M.. (2011). Quantity and quality of exercise for developing and maintaining cardiorespiratory, musculoskeletal, and neuromotor fitness in apparently healthy adults: guidance for prescribing exercise. Med. Sci. Sports Exerc. 43, 1334–1359. 10.1249/MSS.0b013e318213fefb 21694556

[B26] Gentil P. Bottaro M. Noll M. Werner S. Vasconcelos J. C. Seffrin A.. (2017a). Muscle activation during resistance training with no external load - effects of training status, movement velocity, dominance, and visual feedback. Physiol. Behav. 179:4. 10.1016/j.physbeh.2017.06.004 28606773

[B27] Gentil P. Fisher J. Steele J. (2017b). A review of the acute effects and long-term adaptations of single- and multi-joint exercises during resistance training. Sport. Med. 47, 843–855. 10.1007/s40279-016-0627-5 27677913

[B28] Gentil P. Soares S. Bottaro M. (2015). Single vs. multi-joint resistance exercises: effects on muscle strength and hypertrophy. Asian J. Sport. Med. 6:e24057. 10.5812/asjsm.24057 26446291 PMC4592763

[B29] Gentil P. Soares S. R. Pereira M. C. Cunha R. R. Martorelli S. S. Martorelli A. S.. (2013). Effect of adding single-joint exercises to a multi-joint exercise resistance-training program on strength and hypertrophy in untrained subjects. Appl. Physiol. Nutr. Metab. 38, 341–344. 10.1139/apnm-2012-0176 23537028

[B30] Gordon B. R. McDowell C. P. Hallgren M. Meyer J. D. Lyons M. Herring M. P. (2018). Association of efficacy of resistance exercise training with depressive symptoms meta-analysis and meta-regression: analysis of randomized clinical trials. JAMA Psychiatry 75, 566–576. 10.1001/jamapsychiatry.2018.0572 29800984 PMC6137526

[B31] Hardee J. P. Porter R. R. Sui X. Archer E. Lee I. M. Lavie C. J.. (2014). The effect of resistance exercise on all-cause mortality in cancer survivors. Mayo Clin. Proc. 89, 1108–1115. 10.1016/j.mayocp.2014.03.018 24958698 PMC4126241

[B32] Impellizzeri F. M. Marcora S. M. Coutts A. J. (2019). Internal and external training load: 15 years on. Int. J. Sports Physiol. Perform. 14, 270–273. 10.1123/ijspp.2018-0935 30614348

[B33] Kikuchi N. Nakazato K. (2017). Low-load bench press and push-up induce similar muscle hypertrophy and strength gain. J. Exerc. Sci. Fit. 15, 37–42. 10.1016/j.jesf.2017.06.003 29541130 PMC5812864

[B34] Kraemer W. J. Adams K. Cafarelli E. Dudley G. A. Dooly C. Feigenbaum M. S.. (2002). American College of Sports Medicine position stand. Progression models in resistance training for healthy adults. Med. Sci. Sport. Exerc. 34, 364–380. 10.1097/00005768-200202000-00027 11828249

[B35] Loturco I. Nakamura F. (2016). Training periodisation: an obsolete methodology? Aspetar. Sport. Med. J. 5, 110–115.

[B36] Loturco I. Nakamura F. Kobal R. Gil S. Pivetti B. Pereira L.. (2016). Traditional periodization versus optimum training load applied to soccer players: effects on neuromuscular abilities. Int. J. Sports Med. 37, 1051–1059. 10.1055/s-0042-107249 27706551

[B37] MacDonald H. V. Johnson B. T. Huedo-Medina T. B. Livingston J. Forsyth K. C. Kraemer W. J.. (2016). Dynamic resistance training as stand-alone antihypertensive lifestyle therapy: a meta-analysis. J. Am. Heart Assoc. 5:3231. 10.1161/JAHA.116.003231 27680663 PMC5121472

[B38] Martins W. R. de Oliveira R. J. Carvalho R. S. de Oliveira Damasceno V. da Silva V. Z. M. Silva M. S. (2013). Elastic resistance training to increase muscle strength in elderly: a systematic review with meta-analysis. Arch. Gerontol. Geriatr. 57, 8–15. 10.1016/j.archger.2013.03.002 23562413

[B39] Martorelli A. S. de Lima F. D. Vieira A. Tufano J. J. Ernesto C. Boullosa D.. (2020). The interplay between internal and external load parameters during different strength training sessions in resistance-trained men. Eur. J. Sport Sci. 10.1080/17461391.2020.1725646. [Epub ahead of print]. 32008472

[B40] McDonagh M. J. Davies C. T. (1984). Adaptive response of mammalian skeletal muscle to exercise with high loads. Eur. J. Appl. Physiol. Occup. Physiol. 52, 139–155. 10.1007/BF00433384 6370691

[B41] Mitchell C. J. Churchward-Venne T. A. West D. W. D. Burd N. A. Breen L. Baker S. K.. (2012). Resistance exercise load does not determine training-mediated hypertrophic gains in young men. J. Appl. Physiol. 113, 71–77. 10.1152/japplphysiol.00307.2012 22518835 PMC3404827

[B42] Morton R. W. Oikawa S. Y. Wavell C. G. Mazara N. McGlory C. Quadrilatero J. (2016). Neither load nor systemic hormones determine resistance training-mediated hypertrophy or strength gains in resistance-trained young men. J. Appl. Physiol. 121, 129–138. 10.1152/japplphysiol.00154.2016 27174923 PMC4967245

[B43] Muniyappa R. Gubbi S. (2020). COVID-19 pandemic, corona viruses, and diabetes mellitus. Am. J. Physiol. Endocrinol. Metab. 318, E736–E741. 10.1152/ajpendo.00124.2020 32228322 PMC7191633

[B44] Nikolaidis P. T. Del Coso J. Rosemann T. Knechtle B. (2019). Muscle strength and flexibility in male marathon runners: the role of age, running speed and anthropometry. Front. Physiol. 10, 1–9. 10.3389/fphys.2019.01301 31681011 PMC6805725

[B45] Ortega F. B. Silventoinen K. Tynelius P. Rasmussen F. (2012). Muscular strength in male adolescents and premature death: cohort study of one million participants. BMJ 345:e7279. 10.1136/bmj.e7279 23169869 PMC3502746

[B46] Ozaki H. Loenneke J. P. Buckner S. L. Abe T. (2016). Muscle growth across a variety of exercise modalities and intensities: contributions of mechanical and metabolic stimuli. Med. Hypotheses 88, 22–26. 10.1016/j.mehy.2015.12.026 26880629

[B47] Ozaki H. Loenneke J. P. Thiebaud R. S. Abe T. (2015). Cycle training induces muscle hypertrophy and strength gain: strategies and mechanisms. Acta Physiol. Hung. 102, 1–22. 10.1556/APhysiol.102.2015.1.1 25804386

[B48] Paoli A. Gentil P. Moro T. Marcolin G. Bianco A. (2017). Resistance training with single vs. multi-joint exercises at equal total load volume: effects on body composition, cardiorespiratory fitness, and muscle strength. Front. Physiol. 8:e1105. 10.3389/fphys.2017.01105 29312007 PMC5744434

[B49] Paoli A. Moro T. Bianco A. (2014). Lift weights to fight overweight. Clin. Physiol. Funct. Imaging. 35, 1–6. 10.1111/cpf.12136 24612071

[B50] Ruiz J. R. Sui X. Lobelo F. Morrow J. R. Jackson A. W. Sjöström M.. (2008). Association between muscular strength and mortality in men: prospective cohort study. BMJ 337:a439. 10.1136/bmj.a439 18595904 PMC2453303

[B51] Salerno M. Sessa F. Piscopo A. Montana A. Torrisi M. Patanè F.. (2020). No autopsies on COVID-19 deaths: a missed opportunity and the lockdown of science. J. Clin. Med. 9:51472. 10.3390/jcm9051472 32422983 PMC7291342

[B52] Shahid Z. Kalayanamitra R. McClafferty B. Kepko D. Ramgobin D. Patel R.. (2020). COVID−19 and older adults: what we know. J. Am. Geriatr. Soc. 68:e16472. 10.1111/jgs.16472 32255507 PMC7262251

[B53] Singh A. K. Gupta R. Misra A. (2020). Comorbidities in COVID-19: outcomes in hypertensive cohort and controversies with renin angiotensin system blockers. Diabetes Metab. Syndr. Clin. Res. Rev. 14, 283–287. 10.1016/j.dsx.2020.03.016 PMC7144598 32283499

[B54] Souza D. Barbalho M. Gentil P. (2020). The role of resistance training volume on muscle size and lean body mass: to infinity and beyond? Hum. Mov. 21. [Epub ahead of print].

[B55] Souza D. Barbalho M. Vieira C. A. Martins W. R. Cadore E. L. Gentil P. (2019). Minimal dose resistance training with elastic tubes promotes functional and cardiovascular benefits to older women. Exp. Gerontol. 115, 132–138. 10.1016/j.exger.2018.12.001 30550762

[B56] Steele J. Androulakis-Korakakis P. Perrin C. Fisher J. P. Gentil P. Scott C.. (2019). Comparisons of resistance training and ‘cardio' exercise modalities as countermeasures to microgravity induced physical deconditioning: new perspectives and lessons learned from terrestrial studies. Front. Physiol. 10:1150. 10.3389/fphys.2019.01150 31551818 PMC6746842

[B57] Steele J. Fisher J. Giessing J. Gentil P. (2017a). Clarity in reporting terminology and definitions of set endpoints in resistance training. Muscle Nerve 56, 368–374. 10.1002/mus.25557 28044366

[B58] Steele J. Fisher J. Skivington M. Dunn C. Arnold J. Tew G.. (2017b). A higher effort-based paradigm in physical activity and exercise for public health: making the case for a greater emphasis on resistance training. BMC Public Health 17:300. 10.1186/s12889-017-4209-8 28381272 PMC5382466

[B59] Takahashi A. Abe K. Usami K. Imaizumi H. Hayashi M. Okai K.. (2015). Simple resistance exercise helps patients with non-alcoholic fatty liver disease. Int. J. Sports Med. 36, 848–852. 10.1055/s-0035-1549853 26090879

[B60] Takahashi A. Imaizumi H. Hayashi M. Okai K. Abe K. Usami K.. (2017). Simple resistance exercise for 24 weeks decreases alanine aminotransferase levels in patients with non-alcoholic fatty liver disease. Sport. Med. Int. Open 1, E2–E7. 10.1055/s-0042-117875 30539079 PMC6226060

[B61] Tasiopoulos I. Nikolaidis P. T. Tripolitsioti A. Stergioulas A. Rosemann T. Knechtle B. (2018). Isokinetic characteristics of amateur boxer athletes. Front. Physiol. 9:1597. 10.3389/fphys.2018.01597 30487753 PMC6246623

[B62] Tsuzuku S. Kajioka T. Sakakibara H. Shimaoka K. (2017). Slow movement resistance training using body weight improves muscle mass in the elderly: a randomized controlled trial. Scand. J. Med. Sci. Sports, 28, 1339–1344. 10.1111/sms.13039 29247985

[B63] Vale A. F. Carneiro J. A. Jardim P. C. V. Jardim T. V. Steele J. Fisher J. P.. (2018). Acute effects of different resistance training loads on cardiac autonomic modulation in hypertensive postmenopausal women. J. Transl. Med. 16:240. 10.1186/s12967-018-1615-3 30165858 PMC6117915

[B64] Zhao R. Zhao M. Xu Z. (2015). The effects of differing resistance training modes on the preservation of bone mineral density in postmenopausal women: a meta-analysis. Osteoporos. Int. 26, 1605–1618. 10.1007/s00198-015-3034-0 25603795

[B65] Zhou F. Yu T. Du R. Fan G. Liu Y. Liu Z.. (2020). Clinical course and risk factors for mortality of adult inpatients with COVID-19 in Wuhan, China: a retrospective cohort study. Lancet 395, 1054–1062. 10.1016/S0140-6736(20)30566-3 32171076 PMC7270627

